# Perfusionists: The Camouflaged Healthcare Professionals

**DOI:** 10.21470/1678-9741-2022-0474

**Published:** 2023-08-07

**Authors:** P Sainath, V Ladeeda, Lewis Jessica

**Affiliations:** 1 Department of Perfusion Technology, Manipal College of Health Professions, Manipal, Karnataka, India

Dear Editor,

One of the most significant clinical medical breakthroughs during the latter half of the 20^th^ century was the conception and use of cardiopulmonary bypass (CPB) to facilitate open heart surgery. This innovation paved the way for additional developments like extracorporeal circulatory support, ventricular assist devices, mechanical circulatory support, and total artificial hearts. With the ability to bypass both the heart and lungs, surgeons can now fix cardiac abnormalities, replace defunct valves, and bypass occluded coronary arteries^[1]^. Subsequently, there was a demand for people who could operate the new life-supporting devices when open heart procedures with extracorporeal circulation became a reality^[2]^.

Conventional approach has been that a ‘still and bloodless field’ is the ideal setting for cardiothoracic surgery. CPB does this by integrating a pump to take place of the heart and a gas exchange device ‘oxygenator’, which functions as artificial lungs^[3]^. By removing the heart and lungs from circulation, CPB primarily aims to facilitate cardiothoracic procedures while providing adequate gas exchange, systemic organ perfusion, and a way to regulate body temperature. Extracorporeal membrane oxygenation (ECMO) is a therapy that enables the technology associated with CPB to be utilised in intensive care units.

ECMO can substitute the function of the heart and lungs, hence it is seen as a medical approach for both cardiac and respiratory dysfunction^[3]^. It is a life-saving intervention for individuals with refractory, severe respiratory, and circulatory failure. In the current coronavirus disease 2019 (or COVID-19) situation, a vast majority of patients experience acute respiratory distress syndrome, and ECMO is advised in such situations.

Since ECMO is only available in specialised facilities, patients may need to travel for treatment. Critically ill individuals, who are frequently mechanically ventilated, receiving continuous drug infusions and/or receiving extracorporeal circulation treatment, may need to be transported from one facility to another, which is done with the help of an air ambulance. Due to the intricacy, ongoing lack of time, and environmental pressures, transporting patients while they are receiving ECMO support is extremely difficult. Air missions also expose both the patient and the medical team to risks like hypoxia, reduced air humidity, changes in barometric pressures, temperature variations, and the potential to easily become fatigued^[4]^. Exhaustion due to manpower issues — as the workload and the number of patients grow daily with limited number of perfusionists — is also an unnoticed workplace challenge that perfusionists encounter. From the time of initiation of ECMO, a perfusionist must be on duty around-the-clock to monitor the patient parameters and to prevent any unusual events^[5]^. Establishing ECMO for patients infected with contagious diseases puts the perfusionist on risk of contracting the disease. Also, wearing the personal protective equipment kit for eight to 12 hours is quite aggravating and draining. Hence, factors related to job demands like stress, conflict, call responsibilities, working hours, and case load, all have an absolute correlation with burnout. As the necessity for the use of life support devices grows daily, so does the need for perfusionists to run these complex machines. The grim reality is that among all healthcare employees, perfusionists have the least recognition for what they do as they are given the least credit for it and are usually overlooked. People frequently don’t understand what our work entails or how we contribute to healthcare. To address these issues, it can be beneficial to enhance the working environment, acknowledge the value that our industry brings to society, increase the hiring rate, and offer enough economic benefits. By publishing this brief letter, we hope that this profession will emerge from the shadows and demonstrate the actual potential of perfusionists and their dedication to the community.
